# Assessment of the Mandibular Osseous Architecture in Cleft Lip and Palate Using Fractal Dimension Analysis: A Pilot Study

**DOI:** 10.3390/jcm13237334

**Published:** 2024-12-02

**Authors:** Samet Özden, Orhan Cicek

**Affiliations:** 1 Department of Orthodontics, Faculty of Dentistry, İnönü University, Malatya 44280, Türkiye; drsametozden@gmail.com; 2 Department of Orthodontics, Faculty of Dentistry, Zonguldak Bulent Ecevit University, Zonguldak 67600, Türkiye

**Keywords:** bilateral cleft lip and palate, unilateral cleft lip and palate, fractal dimension analysis, metabolic activity, trabeculation

## Abstract

**Background/Objectives**: Although there has been extensive research on the orofacial morphologic effects of cleft lip and palate (CLP), the effects of CLP on mandibular structures remain largely unknown. The aim of this study was to investigate the trabeculation differences in the mandibular osseous architecture of patients with bilateral CLP (BCLP) and left-sided unilateral CLP (UCLP) using fractal dimension (FD) analysis and to compare these findings with healthy controls without CLP. **Methods**: A total of 63 patients (27 females, 36 males) with a mean age of 9.69 ± 1.5 years in the pre-peak growth stage were divided into three groups (n = 21 per group): the control group (CG), the BCLP group, and the UCLP group. The FD analysis was conducted on selected regions of interest (ROIs) from the mandibular condyle, angulus, corpus, and coronoid areas in TIFF-formatted panoramic radiographs. Statistical analyses were performed using the paired t-test and ANOVA for parametric data, and the Wilcoxon and Kruskal–Wallis tests for nonparametric data. Statistical significance was set at *p* < 0.05. **Results**: The FD values obtained from the ROIs of the right condyle were found to be significantly lower in the BCLP group compared to the CG and UCLP groups (*p* < 0.05). Conversely, the FD values for the left condyle were significantly higher in the CG group (*p* < 0.05), while no significant differences were observed between the BCLP and UCLP groups (*p* > 0.05). The FD value of the left condyle in the UCLP group was found to be significantly lower than that of the right condyle (*p* < 0.05). In the CG group, the FD values for both the right and left mandibular condyle and corpus were significantly higher than those for the angulus and coronoid regions; in the UCLP group, only the FD values of the right mandibular condyle and corpus were significantly higher than those for the same regions (*p* < 0.05). **Conclusions**: The reduced FD values in the mandibular condyle of CLP patients during the pre-peak growth stage suggest a loss of trabeculation and lower metabolic activity, while similarly, reduced FD values in the corpus region contribute to delayed tooth eruption timing, likely due to decreased masticatory forces during the intercuspal position and altered occlusal relationships. **Clinical Relevance**: In treating CLP patients, particularly with orthopedic face masks, the reduction in metabolic activities in these areas should be considered to achieve the optimal mandibular growth and development, and dental eruptions during the distribution of force from the chin to the corpus and condyle.

## 1. Introduction

Cleft lip and palate (CLP), one of the most common congenital deformities occurring in approximately 1 in 1000 to 1 in 500 live births, has a considerable impact on quality of life by affecting not only dental development and facial growth but also aesthetic, functional, and psychological aspects [1]. Orthodontic treatments, which are routinely performed with the objective of correcting malocclusions in children and adults, also constitute an important part of the overall multidisciplinary treatment process for individuals with CLP [2]. Considering that each child with CLP has unique needs, expectations, and aspirations, the role of the orthodontist is indeed challenging, as there will be numerous instances requiring orthodontic intervention throughout each CLP child’s first 18 to 20 years of life [2,3].

Numerous studies have been conducted on the genetic and environmental factors involved in the etiology of orofacial clefts, and advances in genetics and molecular biology have further elucidated craniofacial development, leading to the recent identification of several genes associated with CLP, including T-box transcription factor-22, poliovirus receptor- related -1, and interferon regulatory factor-6 [4]. Anatomically, the failure of fusion between the frontonasal and maxillary processes during growth and development leads to cleft lip, while the failure of fusion of the palatal shelves of the maxillary processes can result in clefts of the hard and/or soft palate [5]. Orofacial clefts may present as isolated conditions or in various combinations, and they can be classified as either incomplete clefts, which do not extend to the nasal floor, or complete clefts, characterized by the absence of a connection between the alar base and the medial labial element [5,6].

Early lip and palate repairs using different surgical techniques in patients with complete CLP, while not yet fully elucidated, result in an unfavorable growth pattern of the craniofacial complex [7]. Although bilateral cleft lip and palate (BCLP) represents a more severe form of orofacial clefting compared to unilateral cleft lip and palate (UCLP), maxillofacial development is profoundly affected in both cases, necessitating a multidisciplinary approach in their treatment [8,9,10]. In the presence of cleft lip and palate (CLP), the focus on maxillary correction for addressing skeletal Class III malocclusion due to maxillary deficiency has led to limited evaluation of mandibular development, despite numerous cephalometric studies demonstrating abnormal growth in both the maxilla and mandible [11,12,13]. Additionally, while clockwise rotation of the maxilla has been reported in both BCLP and UCLP individuals, this has not been substantiated in the mandible [12,14,15,16]. Furthermore, it has been reported that mandibular asymmetries associated with vertical maxillary dentoalveolar discrepancy are present in patients with complete UCLP [17,18].

The thickness, sponginess, and anisotropy of trabecular bone, which has a higher metabolic activity than cortical bone, affect its architecture [19,20,21]. Fractal dimension (FD) analysis, which has been widely used to assess changes in bone [20,21,22,23], is a method that evaluates the dimensions and characteristics of complex structures and converts them into simple images and numerical values [21]. The FD analysis method has been utilized in dentistry for various purposes, including evaluating the progression of diseases such as gingivitis and periodontitis [24], examining degenerative changes in the temporomandibular joint (TMJ) [19], detecting fibrous dysplasia and ossifying fibroma [25], identifying mandibular changes caused by sickle cell anemia [26], observing trabecular bone alterations following dental implant procedures [27], and assessing the trabecular structure of mandibular condyles in patients with different dentofacial skeletal patterns [28].

The presence of well-defined trabecular plates and dense bone tissues is indicative of a high FD value, suggesting that the examined structure is denser and more complex [20,21]. In contrast, the loss of trabecular bone that occurs during orthodontic treatment or as a result of increased regional pressure caused by mechanical overload leads to lower FD values, which indicate reduced density and complexity [21,28]. A number of studies have evaluated trabecular bone, which exhibits biological fractal characteristics, using FD analysis in dental panoramic radiographs [28,29,30]. Current literature has shown that FD analysis, which enables the expression of complex structures as numerical data, can be applied to dental panoramic radiographs, serving as a guide to reflect trabecular changes in bone, including in the field of dentistry [21,29,30].

A review of the literature reveals that while numerous studies have been conducted on the diagnosis and treatment planning of CLP, as well as the stability and follow-up of dentofacial skeletal outcomes following treatment, research specifically focused on the mandible remains limited, despite reports highlighting its scarcity [12,13,14]. This situation has necessitated the clarification of differences in mandibular osseous trabeculation between patients with and without CLP, in order to provide clinicians with the insights required for understanding the prognosis of mandibular osseous structures throughout the long-term treatment process of CLP, which extends from birth to adulthood. Thus, elucidating the bone trabeculation in various regions of the mandible through FD analysis will furnish invaluable clinical guidance for monitoring trabeculation differences in the prognosis of mandibular structures, notably the mandibular condyle, coronoid, angular region, and corpus, throughout orthopaedic/orthodontic treatment with facemask (reverse headgear) in CLP patients [11].

It has been shown that trabecular bone, which has a higher metabolic activity than cortical bone, is more suitable for assessing changes and that the FD of trabecular bone exhibits a significant correlation with the physical properties of the bone [31,32]. Furthermore, considering the microscopic and macroscopic changes in the jaw bone caused by occlusal and orthodontic forces, the trabecular bone architecture has been reported to have appropriate load-bearing functions [32,33]. However, no study has been found that reports how trabecular structures are affected in the presence of CLP and how they differ from those without CLP.

Therefore, this study aimed to investigate and compare the differences in mandibular osseous trabeculation among patients with BCLP, UCLP, and healthy controls without CLP using FD analysis on panoramic radiographs. The null hypothesis of the study is that there are no significant differences between the groups in terms of FD values for the measured parameters.

## 2. Materials and Methods

### 2.1. Study Design, Ethics, and Consent

This retrospective study was conducted using panoramic radiographs retrieved from the clinical archives of patients with BCLP and UCLP, who comprised the study groups, and healthy individuals without CLP, who formed the control group; all were referred to the Department of Orthodontics at İnönü University. Ethical approval for the study was obtained from the Non-Interventional Clinical Research Ethics Committee of Zonguldak Bülent Ecevit University with decision number 2024/21-17, dated 2 October 2024. Prior to the commencement of treatment, informed consent forms were duly obtained from all patients and their legal guardians. However, due to the retrospective nature of the study, no further consent was required.

### 2.2. Sample, Groups, and Criteria

The power analysis of the study was performed using G*Power software (version 3.1.9.7; Franz Faul, University of Kiel, Kiel, Germany) based on the previous study by Korkmaz et al. [34]. Accordingly, to achieve an actual power of 95% for the study, with a 5% probability of α error (α err prob) and 95% power (1-β err prob), a minimum sample size of 57 (19 per group) was required (non-centrality parameter λ = 16.7638623 and critical F = 3.1682460). To further strengthen the study’s power, a total of 63 samples were included: 21 in the healthy CG group without CLP (11 females and 10 males, mean age 10.11 ± 1.37), 21 in the UCLP group (9 females and 12 males, mean age 9.26 ± 1.59), and 21 in the BCLP group (7 females and 14 males, mean age 9.81 ± 1.45).

Inclusion criteria for the CG group:

- Skeletal Class III (ANB: <0) with maxillary retrognathia (SNA: <80, A to N ⟂ < 0) and normal mandible (SNB: 80 ± 2);
- No history of trauma;
- No history of orthodontic treatment;
- No permanent tooth absence;
- No history of systemic bone disease;
- No pathology, dysfunction, or any other issues in the temporomandibular joint;
- Pre-peak growth and development stage (PP2=, MP3=);
- High-quality and high-resolution panoramic radiographs in tag image file format (TIFF).

Inclusion criteria for the BCLP and UCLP groups:

- Skeletal Class III (ANB: <0) with maxillary retrognathia (SNA: <80, A to N ⟂ < 0) and normal mandible (SNB: 80 ± 2);
- Presence of complete CLP;
- No surgical procedures other than the initial lip and palate repair;
- No history of orthodontic treatment;
- No permanent tooth absence outside the cleft region;
- No history of systemic bone disease;
- No pathology, dysfunction, or any other issues in the temporomandibular joint;
- Pre-peak growth and development stage (PP2=, MP3=);
- High-quality and high-resolution panoramic radiographs in TIFF format.

Samples that did not meet at least one of the specified criteria were excluded from the study. The samples were identified to be in the pre-peak growth and development stages based on the indicators PP2=, which signifies the equality of the epiphysis and diaphysis of the proximal phalanx of the second finger, and MP3=, indicating the equality of the epiphysis and diaphysis of the middle phalanx of the third finger, on hand–wrist radiographs [35].

### 2.3. Radiographs and Cephalometric Measurements

Panoramic radiographs of the study and control groups were taken using the X-ray machine (Planmeca Proline XC, 00880 Helsinki, Finland) after the bite stick was correctly positioned and the parallelism of the Frankfurt horizontal plane to the floor was confirmed. All panoramic radiographs were obtained using the same device, with a resolution of 0.027 mm pixel size at 66 Kv, 5.0 mA and 18 s. The FD analysis was performed on the panoramic radiographs that satisfied the established inclusion criteria.

**Figure 1:**
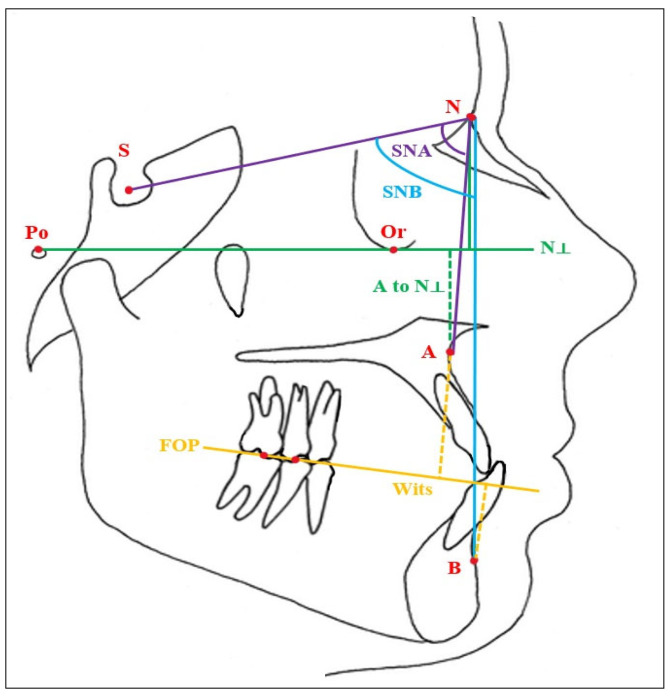
Schematic representation of the cephalometric parameters measured: S: sella, N: nasion, Po: porion, Or: orbitale, A: A point, B: B point, N ⟂: nasion perpendicular, FOP: functional occlusal plane. Red color: Cephalometric landmark points and abbreviations, Purple color: SNA angle, Light blue color: SNB angle, Green color: A to N ⟂ distance, Yellow color: Wits distance.

During the acquisition of lateral cephalometric radiographs, the head position was stabilized using cephalostats for standardization purposes, and the Frankfurt horizontal plane was adjusted to be parallel to the floor. The sagittal skeletal evaluation of the patients’ jaws was performed by measuring the requisite SNA, SNB, and ANB angles using the NemoCeph V.2022 (Nemotec SL, 2006, Madrid, Spain) digital analysis program on lateral cephalometric radiographs obtained from the cephalometric X-ray machine (Planmeca OY, Helsinki, Finland), while the sagittal position of the maxilla was also assessed through the distances from Point A to nasion perpendicular (A to N ⟂, McNamara) and the sagittal distances of Points A and B to the functional occlusal plane (Wits appraisal). The schematic representation of these angular and linear cephalometric measurements is provided in Figure 1. The definitions of the cephalometric measurements are presented in Table 1 [22,36]:

### 2.4. Fractal Analysis

**Figure 2:**
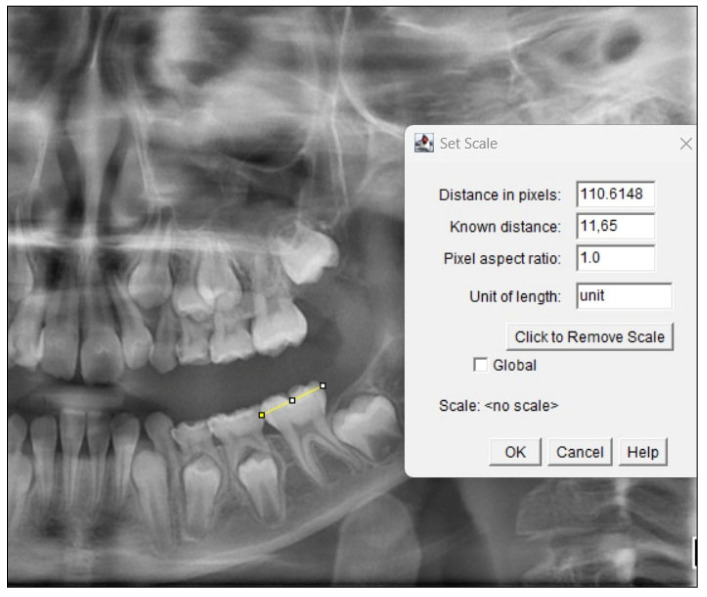
Yellow line: A sample calibrated based on the 11.65 mm mesiodistal distance of the mandibular left permanent first molar.

Fractal dimension analysis was conducted on panoramic radiographs using ImageJ (version 1.53), a Java-based image processing software developed by the National Institutes of Health. The same researcher (SÖ) utilized the same computer (Asus Zenbook UX3402ZA) for FD analysis and employed the box-counting method developed by White and Rudolph [37]. Prior to the FD analysis, the calibration process was conducted on panoramic radiographs obtained in TIFF format to ensure the accuracy and standardization of the measurements in accordance with the specified methodology. The calibration of these panoramic radiographs in TIFF format was performed using the ImageJ software, based on the measurement of the mesiodistal distances of the teeth with a digital caliper (Insize digital caliper, Insize Co., Loganville, GA, USA) on the patients’ orthodontic study casts (see Figure 2).

**Figure 3:**
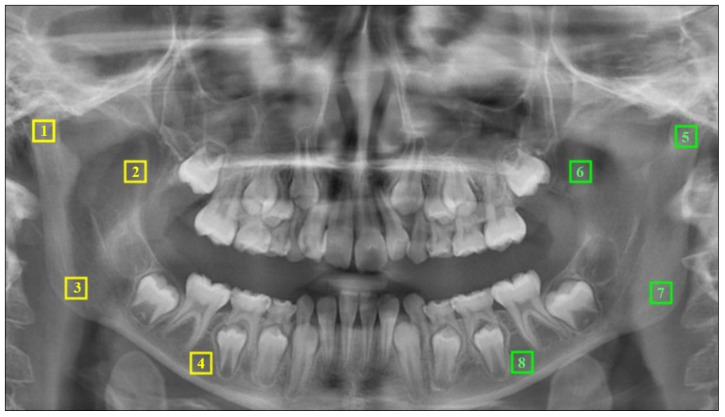
Yellow-colored ROIs for the right mandibular region: (1) condyle, (2) coronoid, (3) angulus, and (4) corpus. Green-colored ROIs for the left mandibular region: (5) condyle, (6) coronoid, (7) angulus, and (8) corpus.

**Figure 4:**
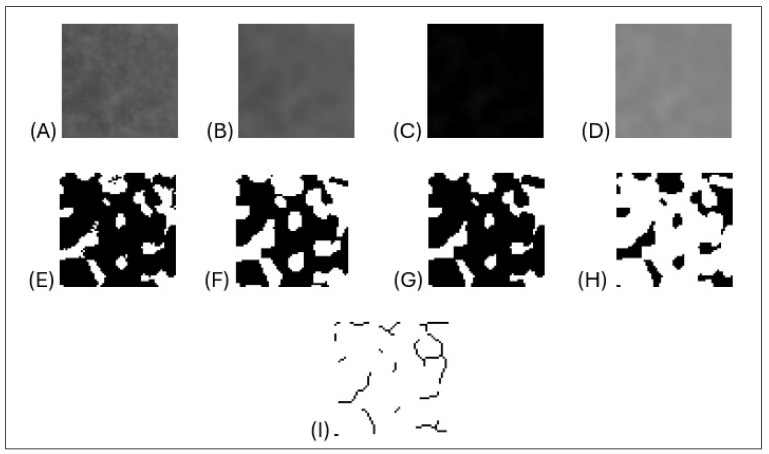
(**A**) Duplicated ROI area image, (**B**) image blurring using the Gaussian filter, (**C**) subtraction of the blurred image from the original image, (**D**) addition of 128 gray tones, (**E**) conversion of the image to black and white using the thresholding method (Make Binary), (**F**) the removal of minor noise from the image via the erode function, (**G**) the enhancement and expansion of the most prominent regions of the image through the use of the dilate function, (**H**) the inversion of colors, and (**I**) the application of the skeletonize method to yield a skeletal appearance.

In the panoramic radiographs of all samples, regions of interest (ROIs) measuring 50 × 50 pixels were identified separately on the right and left sides in the mandibular condyle, coronoid process, angulus, and corpus regions (see Figure 3). Following the selection and saving of the chosen area in 8-bit format, a Gaussian blur filter (sigma = 35 pixels) was applied to the copied image to eliminate factors that cause brightness imbalance, such as soft tissue. By performing a subtraction process with the original image, the resulting image was converted to Binary format by adding 128 gray values. In order to reduce the noise present in the image, a series of operations was applied, beginning with erosion and dilation, followed by the invert option. Subsequently, the skeletonize function was applied to the image with the objective of enhancing the skeletal structure within the bone trabeculae. The final stage of the process was to conduct an FD analysis on the inverted image using the box-counting method [21] (see Figure 4).

### 2.5. Statistical Analysis

Statistical analyses were performed using Statistical Package for Social Sciences version 26 (IBM Co., Armonk, NY, USA). The normality of the data distribution was assessed using the Kolmogorov–Smirnov test. Accordingly, for within-group comparisons of normally distributed dependent data, the paired sample t-test was used, while the Wilcoxon signed-rank test was applied for non-normally distributed data. For comparisons of independent data, the one-way ANOVA (analysis of variance) was used for normally distributed data, with the post hoc Tukey test applied for pairwise comparisons, while for non-normally distributed data, the Kruskal–Wallis test was used, with the post hoc Mann–Whitney U test applied for pairwise comparisons. The chi-square test was applied for categorical variables. To assess intra-observer reliability, we used Cronbach’s α and two-way random effect intra-class correlation coefficients (ICCs) for all measurements in a randomly selected 25% sample. The level of statistical significance was set at *p* < 0.05.

## 3. Results

All repeated measures within each group demonstrated excellent intra-observer reliability, with ICC values ranging from 0.90 to 0.99 (*p* < 0.001).

No significant differences were found between the groups in terms of age and gender among the patients included in the study (*p* > 0.05). The SNA angle was found to be significantly lower in the CG compared to both the BCLP and UCLP groups (*p* < 0.05), with no significant difference observed between the BCLP and UCLP groups (*p* > 0.05). Also, no significant differences were observed between the groups with regard to the SNB angle, ANB angle, A to N ⟂ distance, and Wits distance (*p* > 0.05). Demographic data related to age and gender, as well as angular and linear measurements of cephalometric parameters, are presented in Table 2.

### 3.1. Intra-Group and Inter-Group Comparison Results by Measurement Regions

#### 3.1.1. For Mandibular Condyle

The FD values of the right mandibular condyle were found to be significantly lower in the BCLP group than in the CG and UCLP groups (*p* < 0.05). No significant difference was found between the CG and UCLP groups (*p* > 0.05). The FD values of the left mandibular condyle were found to be significantly higher in the CG compared to the BCLP and UCLP groups (*p* < 0.05), while no significant difference was observed between the BCLP and UCLP groups (*p* > 0.05). A lower FD value was observed in the left condyle compared to the right condyle only in the UCLP group (*p* < 0.05), while no significant differences were seen between the right and left condyle FD values in the other groups (*p* > 0.05).

#### 3.1.2. For Mandibular Angulus

No significant differences were observed between the groups in the FD values of both the right and left mandibular angulus (*p* > 0.05). Similarly, no significant differences were observed between the FD values of the right and left angulus in all groups (*p* > 0.05).

#### 3.1.3. For Mandibular Corpus

No significant differences were observed between the groups in the FD values of both the right and left mandibular corpus (*p* > 0.05). However, in the UCLP group, a lower FD value was observed in the left corpus compared to the right corpus (*p* < 0.05), while no significant differences were noted between the right and left corpus FD values in the other groups (*p* > 0.05).

#### 3.1.4. For Mandibular Coronoid

No significant differences were observed between the groups in the FD values of both the right and left mandibular coronoids (*p* > 0.05). Similarly, no significant differences were observed between the FD values of the right and left coronoids in all groups (*p* > 0.05).

The results of the intra-group and inter-group statistical comparisons of the FD values measured in the mandibular condyle, angulus, corpus, and coronoid regions are presented in Table 3.

**Figure 5:**
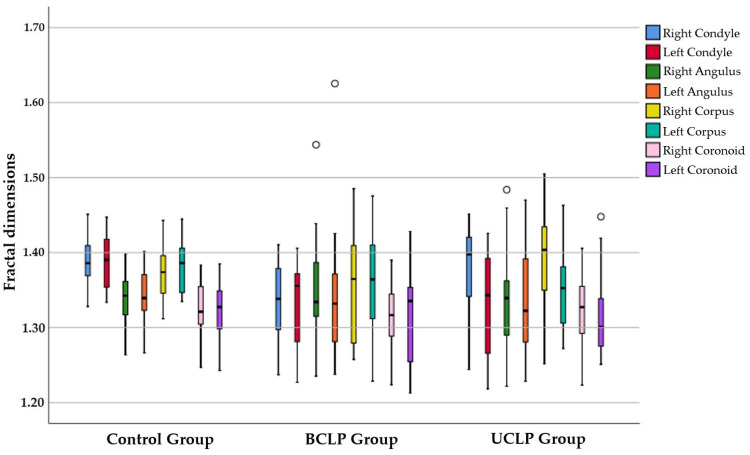
FD values of the right and left mandibular ROIs in the groups.

The box-and-whisker plot illustrating the FD values of the right and left mandibular ROIs across groups is shown in Figure 5.

### 3.2. Intra-Group Comparison Results of Different Regions in the Groups

#### 3.2.1. For CG

The FD values of the mandibular condyle and corpus on both the right and left sides were found to be higher than those of the mandibular angulus and coronoid (*p* < 0.05). However, no significant differences were found between the FD values of the mandibular condyle and corpus, or between the FD values of the mandibular angulus and coronoid (*p* > 0.05).

#### 3.2.2. For BCLP Group

No significant differences were observed between the FD values of the mandibular condyle, angulus, corpus, and coronoid on either the right or left mandibular sides (*p* > 0.05).

#### 3.2.3. For UCLP Group

The FD values of the mandibular condyle and corpus on the right side were found to be higher than those of the mandibular angulus and coronoid (*p* < 0.05). However, no significant differences were found between the FD values of the mandibular condyle and corpus, or between the FD values of the mandibular angulus and coronoid (*p* > 0.05). On the left mandibular side, no significant differences were observed between the FD values of the mandibular condyle, angulus, corpus, and coronoid (*p* > 0.05).

In each group, the statistical comparison results for the mandibular condyle, angulus, corpus, and coronoid are presented in Table 4.

**Figure 6:**
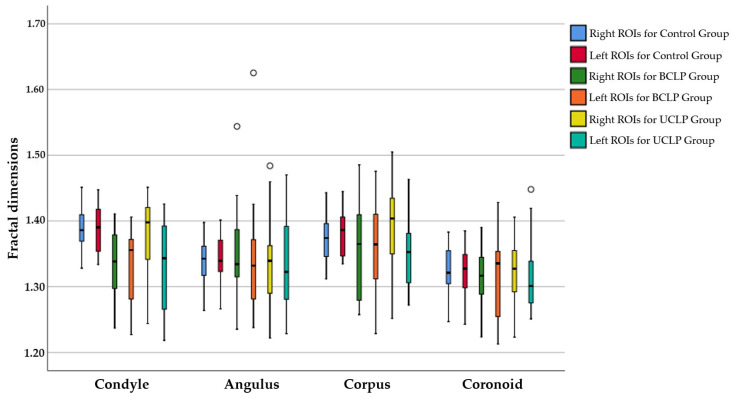
Right and left FD values of groups in the mandibular ROIs.

The box-and-whisker plot illustrating the right and left FD values of the groups for mandibular ROIs is shown in Figure 6.

## 4. Discussion

Various methods, such as micro-computed tomography (micro-CT) [38], magnetic resonance imaging (MRI) [39], histomorphometry [40], dual-energy X-ray absorptiometry (DEXA) [41], intensity features [42], and finite element analysis (FEA) [43], are utilized for evaluating trabecular bone. FD analysis was chosen for this study due to its ability to quantitatively evaluate the microarchitecture of trabecular bone using radiographic images in a fast, cost-effective, and reproducible manner without increasing radiation exposure, making it well-suited for large patient groups.

Fractal dimension analysis has demonstrated utility in various applications, including assessing progression of carcinomas and tumours [44], quantifying trabecular alterations after bone regeneration [45], implant placement [46], evaluating degenerative changes in the TMJ [47], monitoring the recovery of endodontic lesions post-root canal therapy [48], assessing changes in the mandible during functional jaw orthopedics [49] and predicting the success of rapid palatal expansion treatment [50]. Literature generally links a higher complexity in bone structure with increased FD values, whereas lower FD values are associated with a more simplified internal organization [29]. FD analysis can be conducted using patients’ existing dental panoramic radiographs, eliminating the need for additional imaging or materials [51].

Although FD analysis of mandibular bone trabeculation has been extensively studied using panoramic radiographs commonly used in dentistry [22,23,28,29], there is currently no study reporting how mandibular bone fractality is affected in individuals with CLP. In this regard, the present study is the first to compare trabeculation differences in mandibular osseous structures between patients with BCLP and those with UCLP to healthy Class III individuals with similar skeletal characteristics. In addition, the inclusion of pre-peak children of the same age and skeletal characteristics who have not undergone surgical or orthodontic procedures after initial lip and palate repair is a strength of this study, since it minimizes the potential factors that could influence bone trabeculation and, consequently, fractality due to growth and development [52,53].

In the present study, the FD values of the BCLP group, especially in the right mandibular condyle, were found to be significantly lower than those of the CG and UCLP groups. In the left mandibular condyle, the FD values of the CG were found to be significantly higher compared to the other groups. However, in the UCLP group, the FD values of both the mandibular condyle and corpus were found to be lower on the left side compared to the right side. In the ROIs from different areas within the groups, it was observed that the FD values of the right and left mandibular condyles and corpus in the CG were significantly higher compared to those of the mandibular angulus and coronoid, whereas no significant differences were found between the ROIs in the BCLP group. Similarly, in the UCLP group, the FD values of the condyle and corpus were found to be higher in the right mandibular ROIs compared to those of the angulus and coronoid, while no differences were observed among the left mandibular ROIs. These results reveal significant differences in trabeculation of the mandibular condyle and corpus between individuals with and without CLP compared to other regions within the same group and between different groups, leading to rejection of the null hypothesis of the study.

While bone mineral density (BMD) and FD values can be used to determine bone strength [54], it has been suggested that FD analysis allows for a more thorough investigation of biomechanical properties by assessing both the density and strength of the bone [55,56]. As a result, FD analysis has found extensive applications in the field of orthodontics [20,28,29,30,34]. In a study by Afzoon et al. [53] analyzing mandibular condyle trabeculation using FD analysis, significantly less complex trabecular patterns and lower FD values were observed in severe skeletal Class III patients compared to Class I individuals. Conversely, another recent study by Tercanlı et al. [52] investigated the mandibular bone structure of growing children with skeletal Class I, II, and III malocclusions using FD analysis of panoramic radiographs, and the researchers reported that as a result of the study, individuals with skeletal Class I had significantly lower FD values compared to the Class II and III groups, suggesting differences in mandibular trabeculation between different skeletal sagittal patterns. The inconsistency between the results of these two studies may be due to the different age groups within their samples, since Afzoon et al. [53] conducted their study on adults, while Tercanlı et al. [52] focused on growing children. In another study conducted by Nussi et al. [57], using texture analysis on CBCT images, no statistically significant differences were observed between age groups in the trabecular structure of the mandibular condyle. In the present study, to ensure consistent and reliable results, the BCLP and UCLP cleft groups and the non-cleft CG group were composed of samples within the same age range, at the same stage of growth and development, and with identical skeletal characteristics.

In the present study, significant differences in the FD values obtained from the ROIs—particularly in the mandibular condyle and corpus regions—were observed among the non-cleft CG, BCLP, and UCLP groups in children with skeletal Class III malocclusion at the pre-peak stage. It is noteworthy that in healthy children within the CG group, the FD values of the mandibular condyle and corpus were significantly higher compared to other regions; conversely, a decrease was observed in the presence of CLP.

We attribute the higher FD values in the condyle region to the metabolic bone activity associated with growth and development in this area. This situation is consistent with the fact that metabolic bone activity is higher in children than in adults [58]. The researchers have reported that higher FD values may be associated with increased bone metabolism and bone apposition [20], whereas decreased FD values related to decreased metabolic bone activity [59,60]. Additionally, studies have reported a reduction in mandibular bone growth activity in individuals with CLP compared to healthy individuals [12,61]. In our study, consistent with those previous researches, we believe that the higher FD values observed in the mandibular condyle region of healthy controls, compared to cleft patients, are attributable to ongoing growth and development. In a study involving patients with degenerative osteoarthritis, CBCT images were used to examine trabecular structural changes in the condyle. ROIs were selected from the subcortical area of the condyle, revealing that patients with temporomandibular osteoarthritis exhibited lower FD values than those in the healthy control group [47]. This finding suggests that conditions affecting the normal growth and development of the condyle may be associated with reduced FD values. Additionally, another study reported that in growing children treated with functional appliances, FD values in the condylar region decreased by the end of treatment, and this reduction was attributed to newly organized, noncomplex, and unmatured bone density in this area during the treatment process [34].

In our study, similar to results for the condylar region, individuals with CLP were found to have lower FD values in the corpus region compared to healthy individuals. Köse et al. [20] have reported that high FD values indicate faster tooth movement. This finding may help explain the delays in tooth eruption timing observed in individuals with CLP. Furthermore, studies have also indicated delays in mandibular tooth development and eruption in individuals with CLP compared to healthy individuals [62,63,64]. The corpus region is particularly influenced by the occlusal relationships of the premolar and molar teeth, as well as masticatory forces. In their EMG study, Li et al. [65] found that individuals with CLP exhibit lower masseter and temporalis muscle activity in maximum intercuspal position compared to non-cleft individuals, along with longer silent periods in these muscles relative to healthy controls. This finding may help explain the lower FD values observed in the corpus region of individuals with CLP in our study.

The findings emphasize that the observed reduction in FD within the condyle and corpus regions in CLP patients is likely associated with ongoing developmental processes and reduced metabolic activity, rather than pathological conditions requiring intervention. This highlights the importance of taking such physiological variations into account when designing treatment protocols, particularly those involving heavy orthopedic forces, to prevent unintended effects on developing bone structures.

When the four ROI regions were compared among themselves, higher FD values were observed in the condyle and corpus regions compared to the angulus and coronoid regions in non-cleft patients, as well as in the unaffected right side of patients with unilateral cleft (Table 4). This finding suggests higher metabolic activity in the condyle and corpus regions relative to the other two areas. Additionally, masticatory and occlusal forces are transmitted to the condylar region through the corpus, which may contribute to the elevated FD values observed in these two ROI regions. Furthermore, we believe that the decreased FD values in these regions in CLP are also related to decreased metabolic bone activity [59,60]. Moreover, this is further supported by previous studies reporting a deceleration in mandibular growth [12,61] and delays in mandibular tooth development and eruption [62,63,64] in the presence of CLP.

Although the study provides important clinical insights for orthodontists regarding mandibular osseous trabeculation in patients with CLP, it has some limitations: the inability to perform long-term growth and developmental follow-up of these patients—due to ethical reasons, it is difficult to find an untreated adult control group—and the lack of control over the effects of subsequent surgical procedures and orthodontic/orthopeadic interventions on mandibular trabeculation. Another limitation of this study is that the calculation of trabecular differences was based on the analysis of panoramic radiographs, which may not be the ideal method for evaluating skeletal structures because of their two-dimensional nature and the potential for image distortion. Future studies in CLP patients may benefit from the use of advanced imaging modalities such as CBCT, which provides three-dimensional data and allows for a more accurate assessment of the trabecular architecture. However, the study provides significant findings, with a high power analysis ratio, regarding the trabeculation of the mandibular condyle, angulus, corpus, and coronoid regions in healthy controls without CLP and in children with CLP, where the same skeletal standardization was achieved.

## 5. Conclusions

The null hypothesis of the study was rejected. The findings highlight the need to consider physiological variations in CLP patients, such as reduced FD due to developmental processes and metabolic activity, when designing treatment protocols to avoid unintended impacts on developing bone structures. The following conclusions were reached:

- The lower FD values observed in the condyle region of CLP patients emphasize reduced metabolic bone activity and the presence of more immatured bone formation. This finding aligns with the understanding that CLP impacts normal bone development, distinguishing it from the typical growth patterns seen in healthy individuals.
- The lower FD values observed in the corpus region of individuals with CLP highlight reduced bone development and delayed tooth eruption timing, likely due to decreased masticatory forces during intercuspal position and altered occlusal relationships. These findings further differentiate the bone growth patterns in CLP patients from those in healthy individuals.
- Finally, it was concluded that (i) the pattern of endochondral ossification of the condyle should be monitored in patients with CLP, and (ii) when treating CLP patients with facemasks, the distribution of orthopedic forces from the chin to the mandibular corpus and condyle should be taken into account, and the reduced trabeculation in these regions should be considered to ensure the continuity of normal mandibular growth and development.

## Figures and Tables

**Table 1:** Cephalometric parameters and definitions.

Parameter	Definition
**SNA**	The angle between the sella-nasion and nasion A point line, for the anteroposterior position of the maxilla to the cranial base.
**SNB**	The angle between the sella-nasion and nasion B point line, for the anteroposterior position of the mandible to the cranial base.
**ANB**	The angle between the nasion A point and the nasion B point line, anteroposterior position of the maxilla and mandible relative to each other.
**A to N ⟂**	The sagittal distance between the nasion perpendicular line and the vertical projection of point A onto this line.
**Wits Appraisal**	The sagittal distance between the projections of the perpendiculars drawn from points A and B to the functional occlusal plane.

**Table 2:** Statistical results regarding demographic and skeletal angular and linear data in the groups.

		CG ^a^	BCLP ^b^	UCLP ^c^	*p*
Age (year)	Mean ± SD	10.11 ± 1.37	9.81 ± 1.45	9.26 ± 1.59	0.066 ^K^
Gender	Female, n (%)	11 (52)	7 (33)	9 (43)	0.332 ^χ2^
	Male, n (%)	10 (48)	14 (67)	12 (57)	
SNA (°)	Mean ± SD	79.01 ± 4.11 ^b.c^	75.05 ± 3.6	76.31 ± 2.17	0.001 * ^K^
SNB (°)	Mean ± SD	81.71 ± 4.66	79.01 ± 3.23	80.24 ± 2.49	0.057 ^A^
ANB (°)	Mean ± SD	−2.8 ± 1.61	−3.97 ± 1.59	−3.97 ± 2.42	0.103 ^K^
A to N ⟂ (mm)	Mean ± SD	−1.5 ± 3.2	−2.69 ± 3.75	−2.37 ± 3.01	0.56 ^K^
Wits Appraisal (mm)	Mean ± SD	−5.92 ± 3.37	−5.12 ± 2.09	−4.67 ± 3.08	0.372 ^A^

SD: standard deviation, N ⟂: nasion perpendicular, n: sample, %: percentage; CG: control group, BCLP: bilateral cleft lip and palate group, UCLP: unilateral cleft lip and palate group; ^K^: Kruskall– Wallis test, ^χ2^: chi-square test, ^A^: one-way ANOVA (analysis of variance) test, *: *p* < 0.05. ^a^ Difference with CG in the same row *p* < 0.05; ^b^ difference with BCLP in the same row *p* < 0.05; ^c^ difference with UCLP in the same row *p* < 0.05.

**Table 3:** Statistical analysis results for intra-group and inter-group comparisons.

			CG ^a^	BCLP ^b^	UCLP ^c^	*p*
Condyle	Right	Mean ± SD	1.387 ± 0.034	1.332 ± 0.055 ^a,c^	1.379 ± 0.057	0.001 * ^A^
	Left	Mean ± SD	1.389 ± 0.36 ^b,c^	1.33 ± 0.058	1.336 ± 0.066	0.001 * ^K^
	Intra-group difference *p*		0.712 ^P^	0.945 ^W^	0.009 * ^P^	
Angulus	Right	Mean ± SD	1.34 ± 0.034	1.345 ± 0.069	1.335 ± 0.07	0.848 ^A^
	Left	Mean ± SD	1.34 ± 0.038	1.34 ± 0.086	1.34 ± 0.073	0.993 ^A^
	Intra-group difference *p*		0.924 ^P^	0.917 ^W^	0.846 ^P^	
Corpus	Right	Mean ± SD	1.376 ± 0.037	1.357 ± 0.073	1.395 ± 0.07	0.137 ^A^
	Left	Mean ± SD	1.381 ± 0.034	1.358 ± 0.07	1.351 ± 0.053	0.188 ^A^
	Intra-group difference *p*		0.283 ^P^	0.951 ^P^	0.006 * ^P^	
Coronoid	Right	Mean ± SD	1.326 ± 0.036	1.318 ± 0.047	1.321 ± 0.051	0.858 ^A^
	Left	Mean ± SD	1.325 ± 0.039	1.316 ± 0.063	1.317 ± 0.054	0.834 ^A^
	Intra-group difference *p*		0.922 ^P^	0.83 ^P^	0.779 ^P^	

SD: standard deviation, ^A^: one-way ANOVA (analysis of variance) test, ^P^: paired-t test, ^K^: Kruskall– Wallis test, ^W^: Wilcoxon signed ranks test, *: *p* < 0.05, CG: control group, BCLP: bilateral cleft lip and palate group, UCLP: unilateral cleft lip and palate group. ^a^ Difference with CG in the same row *p* < 0.05; ^b^ difference with BCLP in the same row *p* < 0.05; ^c^ difference with UCLP in the same row *p* < 0.05.

**Table 4:** Statistical analysis results for regional comparisons within the group.

			Condyle ^a^	Angulus ^b^	Corpus ^c^	Coronoid ^d^	*p*
For CG	Right	Mean ± SD	1.387 ± 0.034 ^b,d^	1.34 ± 0.034	1.376 ± 0.037 ^b,d^	1.326 ± 0.036	<0.001 * ^A^
	Left	Mean ± SD	1.389 ± 0.36 ^b,d^	1.34 ± 0.038	1.381 ± 0.034 ^b,d^	1.325 ± 0.039	<0.001 * ^A^
For BCLP	Right	Mean ± SD	1.332 ± 0.055	1.345 ± 0.069	1.357 ± 0.073	1.318 ± 0.047	0.216 ^A^
	Left	Mean ± SD	1.33 ± 0.058	1.34 ± 0.086	1.358 ± 0.07	1.316 ± 0.063	0.268 ^A^
For UCLP	Right	Mean ± SD	1.379 ± 0.057 ^b,d^	1.335 ± 0.07	1.395 ± 0.07 ^b,d^	1.321 ± 0.051	<0.001 * ^A^
	Left	Mean ± SD	1.336 ± 0.066	1.34 ± 0.073	1.351 ± 0.053	1.317 ± 0.054	0.306 ^K^

SD: standard deviation, n: sample, ^A^: one-way ANOVA (analysis of variance) test, ^K^: Kruskall–Wallis test, *: *p* < 0.05, CG: control group, BCLP: bilateral cleft lip and palate group, UCLP: unilateral cleft lip and palate group. ^a^ Difference with condyle in the same row *p* < 0.05; ^b^ difference with angulus in the same row *p* < 0.05; ^c^ difference with corpus in the same row *p* < 0.05; ^d^ difference with coronoid in the same row *p* < 0.05.

## Data Availability

All data supporting the results of this study are included within the study.
